# P-1746. Evaluating Microbiology Reporting on Time to Appropriate Therapy for Organisms Producing Extended-spectrum and AmpC β-lactamases

**DOI:** 10.1093/ofid/ofae631.1909

**Published:** 2025-01-29

**Authors:** Alison Samsel, Jennifer Kelly, Nicole Harrington, Kelly Curran, Julie Ing, Clint Borja

**Affiliations:** ChristanaCare, Newark, Delaware; ChristianaCare, Newark, Delaware; ChristianaCare, Newark, Delaware; ChristianaCare, Newark, Delaware; ChristianaCare, Newark, Delaware; ChristianaCare, Newark, Delaware

## Abstract

**Background:**

Our Antimicrobial Stewardship Program (ASP) implemented specific treatment recommendations and suppression of select susceptibility results for microbiology reports positive for extended-spectrum β-lactamases (ESBL) and AmpC producing organisms. These statements also recommend Infectious Diseases (ID) consult if needed. This change is in alignment with the Clinical and Laboratory Standards Institute (CLSI) recommendations.

**Figure 1:**
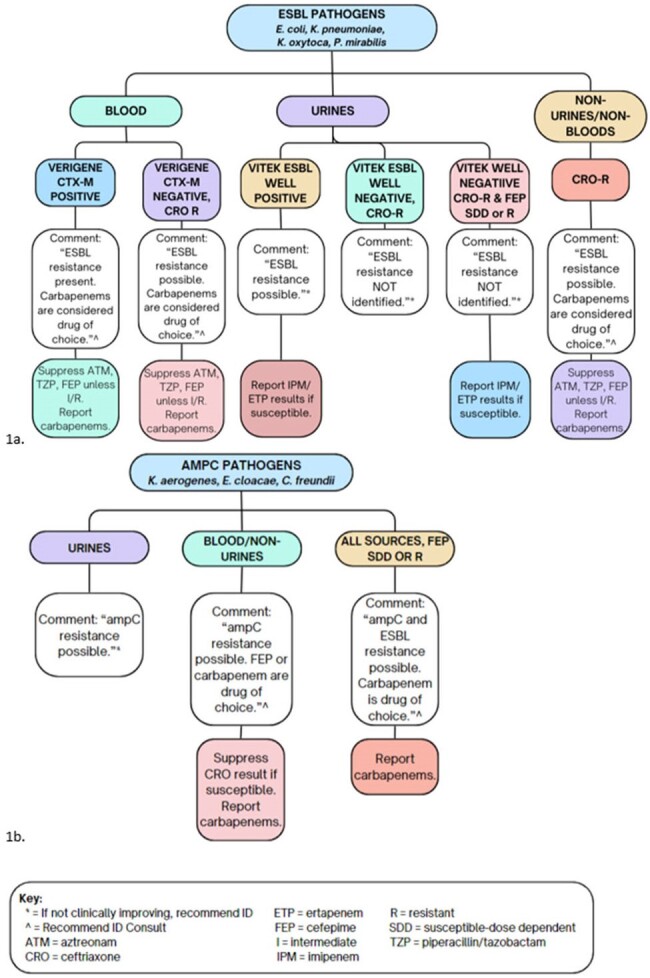
Microbiology lab reporting statements A. ESBL organism statements. B. AmpC organism statements.

**Methods:**

This study was a single-center, retrospective cohort of adult patients with monomicrobial infections due to organisms at high-risk for AmpC or ESBL production. The intervention was effective February 28th, 2023. See Figure 1. Data was collected by chart review for pre- and post-intervention groups from April 1st to September 30th, 2022, and April 1st to September 30th, 2023, respectively. The primary outcome was the time to appropriate definitive antibiotic administration. Appropriate antibiotic therapy was defined by the Infectious Diseases Society of America (IDSA) 2023 Guidance on the Treatment of Antimicrobial Resistant Gram-Negative Infections guidelines. Secondary outcomes included guideline-adherent antibiotic therapy, frequency of ID consultations, and inappropriate antibiotic de-escalation.

**Results:**

Time to appropriate antibiotic administration was not significantly different between the pre- and post-intervention group (median time 8.6 hours vs. 8.3 hours, respectively). The frequency that patients received guideline-adherent therapy significantly increased between groups (pre- 67% vs post- 81%, p = 0.024). ID consultations also significantly increased (pre- 52% vs post- 77%, p = 0.005). Inappropriate de-escalation of therapy based on susceptibilities significantly decreased between groups (pre- 12% vs post- 2%, p = 0.047).

**Conclusion:**

The implementation of microbiology reporting statements increased guideline-adherent definitive antibiotic therapy, increased ID consultations and decreased inappropriate antibiotic de-escalation.

**Disclosures:**

**All Authors**: No reported disclosures

